# Supplementary material for: Antifungal Properties of Biogenic Selenium Nanoparticles Functionalized with Nystatin for the Inhibition of Candida albicans Biofilm Formation
Source: Molecules. 2023 Feb 15;28(4):1836. doi: 10.3390/molecules28041836 (PMC9958786; doi:10.3390/molecules28041836)
Supplement: Supplementary file 1 [file molecules-28-01836-s001.zip › molecules-2145515-supplementary.pdf]

Supplementary materials

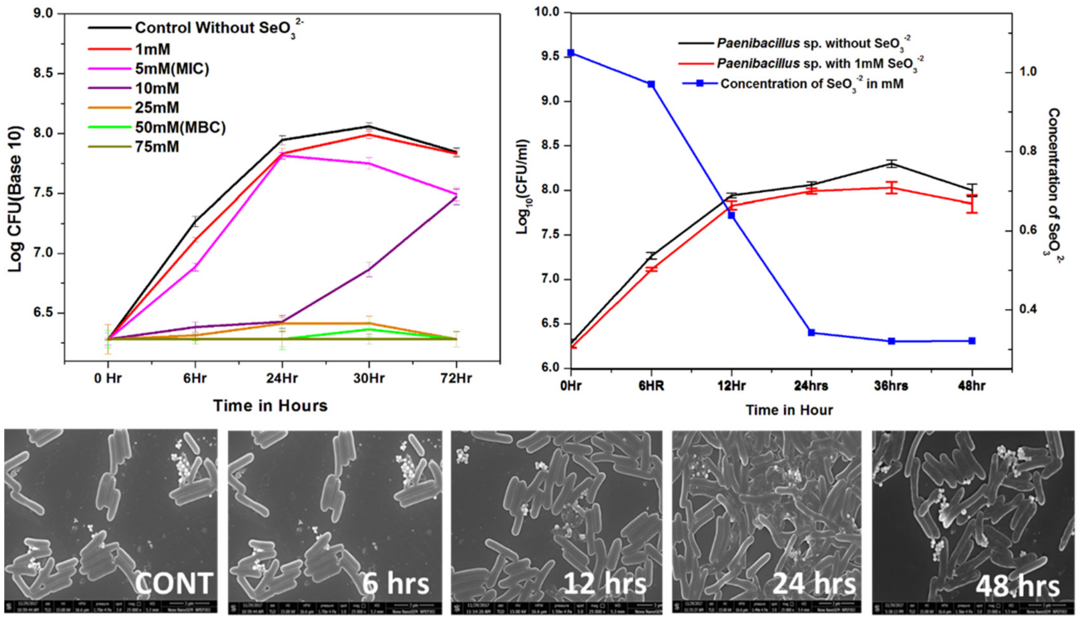

Figure S1. The growth kinetics of P. terreus in different concentration of sodium selenite shown in log cfu (left). Growth of *P. terreus* in presence of 1mM sodium selenite and corresponding decrease in the concentration of selenium ions after synthesizing SeNPs (Z-axis). Lower panel indicate the FESEM micrographs showing the size of SeNPs and cells during the synthesis biogenic SeNPs.

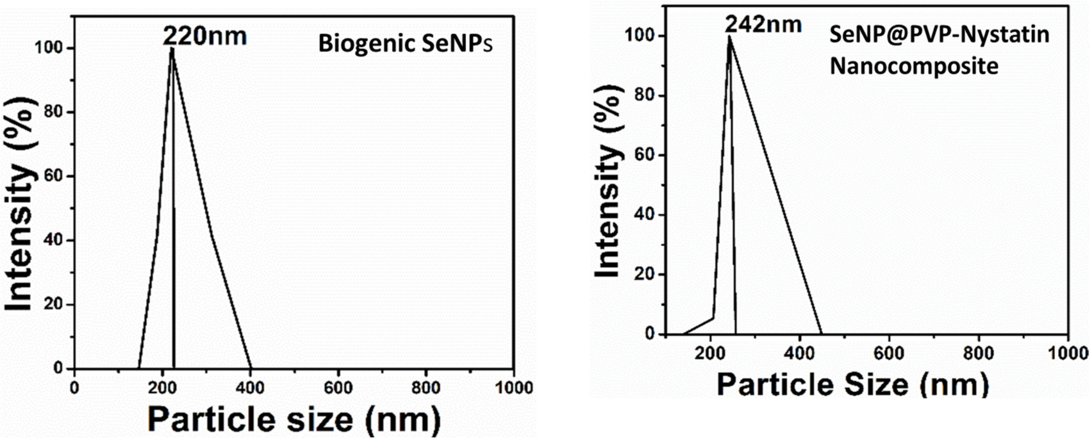

Figure S2. DLS analysis of Biogenic SeNPs (left) and SeNP@PVP-Nystatin nanoconjugates (right) showing the particle size distribution of biogenic SeNPs and nanoconugates. The average particle size of biogenic SeNPs and nanoconugates was respectively, 220 ηm and 242 ηm.

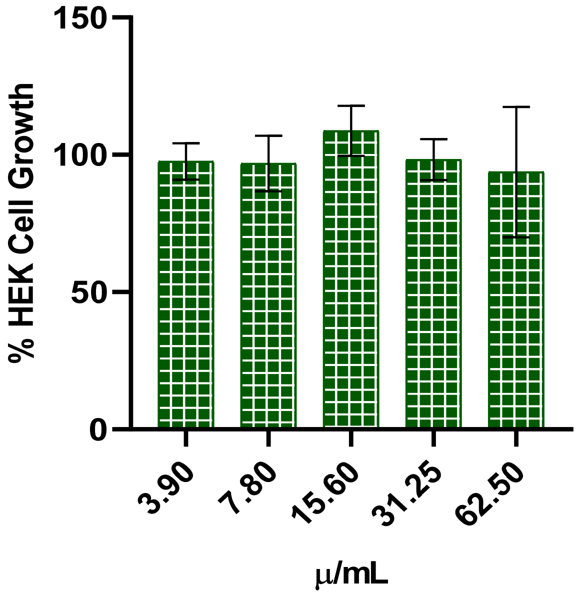

Figure S3. Cytotoxicity study of SeNP@PVP-Nystatin nanoconjugates on HEK-293 cell line.
